# Periodic, *n*-soliton and variable separation solutions for an extended (3+1)-dimensional KP-Boussinesq equation

**DOI:** 10.1038/s41598-023-42845-0

**Published:** 2023-09-22

**Authors:** Chuanlin Shao, Lu Yang, Yongsheng Yan, Jingyu Wu, Minting Zhu, Lingfei Li

**Affiliations:** 1 School of Economics and Finance, Huaqiao University, Quanzhou, 362021 Fujian People’s Republic of China; 2 School of Economics and Management, Northwest University, Xi’an, 710127 Shaanxi People’s Republic of China

**Keywords:** Applied mathematics, Fluid dynamics

## Abstract

An extended (3+1)-dimensional Kadomtsev–Petviashvili–Boussinesq equation is studied in this paper to construct periodic solution, *n*-soliton solution and folded localized excitation. Firstly, with the help of the Hirota’s bilinear method and ansatz, some periodic solutions have been derived. Secondly, taking Burgers equation as an auxiliary function, we have obtained *n*-soliton solution and *n*-shock wave. Lastly, we present a new variable separation method for (3+1)-dimensional and higher dimensional models, and use it to derive localized excitation solutions. To be specific, we have constructed various novel structures and discussed the interaction dynamics of folded solitary waves. Compared with the other methods, the variable separation solutions obtained in this paper not only directly give the analytical form of the solution *u* instead of its potential $u_y$, but also provide us a straightforward approach to construct localized excitation for higher order dimensional nonlinear partial differential equation.

## Introduction

The history of the study of nonlinear evolution equations(NEEs) is sporadic during the past decades. In spit of the fact that physical phenomena have been carried out by the solutions of fundamental nonlinear models, there are a small group of methods can be devised to derive them. NEEs that display wave phenomena can be basically sorted out as dispersive and hyperbolic. The theory of hyperbolic partial differential equation is well formulated, whereas the theory of dispersive wave equation is not, such as the nonlinear Schrödinger equation, sine-Gordon equation, KdV equation as well as the mKdV equation. The application of the NEEs which got the most notice is in fluid dynamic. Lately, the application of the NEEs in other fields, such as water wave, quantum mechanic, crystal optic, lattice dynamic, nerve pulse propagation, active transmission line and various areas of continuum mechanic, are getting more attention and there is an urgent demand for more universal method. Therefore, to derive the analytical solution of NEEs, numerous helping methods have been put forth, such as inverse scattering transform^1^, Darboux transform^2^, Lax pair^3^, Lie group^4^, Hirota bilinear form^5^ as well as Bäcklund transform^6^. Theoretical progress of these nonlinear models depends mainly on how fast one can produce approximate and numerical solution which is sample as much of the corresponding parameter space. In most cases, numerical solution is a poor mean of sampling parameter space to extract the solution and the accuracy of the approximate solution relies on the value of the parameter, large or small. Apart from linearization, other result mainly obtained from the perturbation method.

Rogue wave^7^ has received considerable attentions in recent years. Oceanographers refer to it as a type of isolated, Gaussian-distributed event that occurs in the ocean more frequently than usual. “New Year Wave”^8^, which was discovered in the North Sea on January 1, 1995, is the most well-known rogue wave. The optical rogue wave was first detected through an experiment in 2007^9^ and the ”Peregrine” soliton in 2010^10^. In nature, rogue waves are common and can be found in a variety of settings, including doped fibers^11,12^, acoustic turbulence^13^, microwave transport^14^, nonlinear optical cavities^15^ and finance^16^. The usual property characterizing the rogue wave in different contents is the observance of great deviation of the wave height from Gaussian statistic, along with the probability density function which results in frequent emission of the giant wave. In spite of the distinctiveness of each experiment, other features can also be identified as the existence of the activity of uncorrected “grain”, which distributes in larger spatial domain unevenly. Based on the nature of the wave, grain may stem from different origins, such as the soliton in nonlinear system, packet in linear propagating wave, wave clustering in spatial domain. All of these can occur under different dynamics, as a spatial symmetry breaking, a hypercycle amplification^17^, a temporal delay or a transport phenomenon. On the other hand, it has been proved that rogue wave can be generated in the microwave experiment without the nonlinearity^18^. This implies that the nonlinearity plays the part of inducing soliton-like structure. In order to verify this hypothesis, the researchers conduct a linear optical experiment and compare the result with those from a nonlinear cavity^19^. The outcome indicates the rogue wave relates to the appearance of a suitable amount of inhomogeneity and granularity, independently if they are induced by a linear or nonlinear system.

In real life, most of the waves interact with others. During the interactions , plenty of localized excitation phenomena will arise. The soliton is the most basic excitation of the (1+1)-dimensional nonlinear model, such as peakon, breather, compacton, instanton, kink soliton and bell soliton. In general, there are several “variable separation” methods used to construct folded(multi-valued) waves. The first method is the “formal variable separation approach” (FVSA)^20^; The second method is to find derivative-dependent function separation solution^21^ through conditional symmetry; The third one is the so-called “multi-linear variable separation approach” (MLVSA)^22^. Except for that, ETM^23^ and projective Riccati equation method(PREM)^24^ are also effective in dong that. One common ground among these methods is the existence of free functions. The existence of arbitrary functions render various localized excitations, and the richness of localized excitations plays an essential role in exploring the corresponding dynamical properties of nonlinear models. Dai^25^ has derived exponential variable separation solution for the (2+1)-dimensional KdV equation by the two function approach and discussed the inelastic interactions between foldon and semi-foldon under suitable function selection. Besides, Dai^26^ has derived ten different variable separation solutions for the (2+1)-dimensional Bogoyavlenskii–Schiff model through the generalized Riccati equation mapping and improved projective Riccati equation method that link independent functions. In^27^, Huang analyzed three types of localized excitations: multi-lump, multi-dromion, periodic solution in terms of Hirota’s bilinear method and variable separation approach for an extended Jimbo-Miwa equation. Other (2+1)-dimensional models, for instance, asymmetric NNV equation, the Nizhnik-Novikov-Veselov system, asymmetric DS equation, a general (M+N)-component Ablowitz–Kaup–Newell–Segur system, dispersive long wave equation, Maccari system, Broer–Kaup–Kupershmidt system, long wave–short wave interaction model are considered by Lou^28–30^ and an usual expression with arbitrary functions are established. For more details about variable separation method, the readers can refer to^31–33^.

Until now, the attention is mainly focus on (2+1)-dimensional models while (3+1)-dimensional cases are rarely considered. Beyond that, the localized excitations obtained via the MLVSA needs rigorous prerequisite conditions. Taking (2+1)-dimensional case as an example

1 $$\begin{aligned} \mathcal {N}(u,u_x,u_y,u_t,u_{xy},u_{xt},u_{yt},...)=0. \end{aligned}$$

One has to convert (Eq. 1) into bilinear form at first, via the transformation $u=c_0(\ln f)_x+u_0$ or $u=c_0(\ln f)_{xx}+u_0$, where $c_0$ is a constant and $u_0$ is a function relating to *x*, *y*, *t* which will be determined later. Through long and tedious calculation, its potential $u_y$ usually has the form

$$\begin{aligned} u_y=c_0\frac{\partial }{\partial x}\bigg (\ln \big (a_0+a_1F[x]+a_2G[y,t]+a_3F[x]G[y,t]\big )\bigg )+u_0, \end{aligned}$$

or

$$\begin{aligned} u_y=c_0\frac{\partial ^2}{\partial x^2}\bigg (\ln \big (a_0+a_1F[x]+a_2G[y,t]+a_3F[x]G[y,t]\big )\bigg )+u_0, \end{aligned}$$

where $c_0,a_1,a_2,a_3,a_4$ are constants and $u_0$ might be the function with respect to *x*, *t*. If $u_0$ is truly a function only relates to *x* and *t*, the potential $u_y$ will win its freedom via the choosing of *F*, *G*. Briefly speaking, *F*[*x*] and *G*[*y*, *t*] can be defined to be any functions. However, the above conditions are too rigorous. Particularly, one has to use $u_y$ to simulate *u* and this may cover the true face for what *u* really is. Here, we are stroke by some interesting ideas: Can we relax the constraints? Can we derive variable separation solution in a more simple way? To answer above question, we present a new variable separation solution for (3+1)-dimensional case, i.e.

2 $$\begin{aligned} u=\lambda _1 \digamma _1[x]+\lambda _2 \digamma _2[y]+\lambda _3 \digamma _3[z]+\lambda _4 \digamma _4[t], \end{aligned}$$

where $\lambda _i(i=1,...,4)$ are constants to be determined later, $\digamma _1[x],\digamma _2[y],\digamma _3[z],\digamma _4[t]$ are free functions.

Next, via the ansatz and variable separation function (2), we are going to study a new (3+1)-dimensional generalized KP-Boussinesq equation

3 $$\begin{aligned} u_{xxxy}+3(u_x u_y)_x+u_{tx}+u_{ty}+u_{tt}-u_{xz}=0, \end{aligned}$$

where $u=u(x,y,z,t)$ is differentiable function in terms of temporal variable *t* and spatial variables *x*, *y*, *z*; $u_{xz}$ is an extra item added to KP-Boussinesq equation^34^.

Our treatment goes as follows. In “Painlevé analysis”  , we have transferred Eq. (3) into bilinear form through the logarithmic transformation. Then, with the help of the ansatz that consists of exponential and trigonometric function, periodic solutions have been obtained under specific condition. Moreover, we have plotted three typical periodic solutions in 2D and 3D. In “Periodic solution of (3+1)-dimensional KP-Boussinesq equation” , taking Burgers equation as an auxiliary function, we have derived *n*-soliton as well as *n*-shock wave for Eq. (3). The mechanism of the shock wave have been studied systemically. In “Multi-soliton and shock wave of (3+1)-dimensional KP-Boussinesq equation” , we have used the variable separation function (2) to construct folded solitary waves of Eq. (3) to verify its efficiency. Besides, we have discussed the interaction behaviours and superimposed structures of folded solitary waves. Therefore, lots of new structures have been obtained. To our knowledge, these solutions have not been reported elsewhere. “Variable separation solution of (3+1)-dimensional KP-Boussinesq equation”   contains a summary. The variable separation solutions obtained here not only present the analytical form of solution *u* instead of its potential $u_y$, but also provide us a distinct way to construct localized excitation for higher order dimensional nonlinear partial differential equation. Therefore, we believe the method proposed in current work will be helpful in finding localized excitation solution for other nonlinear systems.

## Painlevé analysis

The integrability of some reductions to ordinary differential equations and the inverse scattering transform integrability of the soliton equation are fundamentally related, as shown by the Painlev’e property. If all of the movable singularity maifolds’ solutions are single-valued, the PDE is said to have passed the Painlev’e test. Under this sense, we follow the same manners of WTC-Kruskal algorithm in^35^. Assuming the solution of Eq. (3) can be expressed as Laurent series

4 $$\begin{aligned} u(x,y,z,t)=\sum \limits ^{\infty }_{j=0}u_{j}(x,y,z,t)\phi (x,y,z,t)^{(j+\alpha )}. \end{aligned}$$

Substitution into Eq. (3) and balancing the most dominant terms, one has

5 $$\begin{aligned} \alpha =-1,\quad u_0(x,y,z,t)=2\left( \frac{\partial }{\partial x}\phi (x,y,z,t)\right) . \end{aligned}$$

Substituting Eqs. (5), (4) into Eq. (3) yields

$$\begin{aligned} \displaystyle u(x,y,z,t)=\frac{2\phi ^2_x}{\phi }+u_1, \end{aligned}$$

and a characteristic equation with one branch for resonances at $r=-1,1,4$ and 6. Applying the Kruskal’s gauge to $\phi (x,y,z,t)=x-\psi$, one has

$$\begin{aligned} \begin{array}{lll} \displaystyle u_0=&{}\displaystyle 2,\\ \displaystyle u_2=&{}\displaystyle \frac{1}{6}\frac{\psi ^2_{t}+\left( \psi _{y}-1\right) \psi _{t}-\psi ^2_{z}-\psi _{z}+3 u_1}{\psi _{y}},\\ \displaystyle u_3=&{}\displaystyle \frac{1}{24\psi ^3_{y}}\left( \left( -\psi ^2_{t}+\psi ^2_{z}+\psi _{t}+\psi _{z}-3u_{1y}\right) \psi _{yy}+2\left( \left( \psi _{t}+\frac{3}{2}\psi _{y}-\frac{1}{2}\right) \psi _{yt}\right. \right. \\ &{}\displaystyle +\left. \left. \left( -\psi _{z}-\frac{1}{2}\right) \psi _{yz}+\psi _{tt}\psi _{y}-\psi _{zz}\psi _{y}+\frac{3}{2}u_{1yy}\right) \psi _{y}\right) ,\\ \displaystyle u_5=&{}\displaystyle \frac{1}{288\psi ^4_{y}}\left( 9\left( \frac{1}{3}\psi ^2_{t}-\frac{1}{3}\psi _{t}\psi _{y}-\frac{1}{3}\psi ^2_{z}+u_{1y}-\frac{1}{3}\psi _{t}-\frac{1}{3}\psi _{z}\right) \left( \frac{1}{3}\psi ^2_{t}-\frac{1}{3}\psi ^2_{z}+u_{1y}-\frac{1}{3}\psi _{t}-\frac{1}{3}\psi _{z}\right) \psi _{yy}\right) \\ &{}\displaystyle +6\left( \left( \frac{1}{2}\psi _{t}\psi ^2_{y}+\left( \frac{1}{6}\psi ^2_{t}+\frac{1}{6}\psi ^2_{z}+\frac{1}{6}\psi _{z}-\frac{1}{2}u_{1y}\right) \psi _{y}-3\left( \psi _{t}-\frac{1}{2}\right) \left( \frac{1}{3}\psi ^2_{t}-\frac{1}{3}\psi ^2_{z}+u_{1y}-\frac{1}{3}\psi _{t}-\frac{1}{3}\psi _{z}\right) \right. \psi _{yt}\right. \\ &{}\displaystyle +3\left( \psi _{z}+\frac{1}{2}\right) \left( \frac{1}{3}\psi ^2_{t}-\frac{1}{9}\psi _{t}\psi _{y}-\frac{1}{3}\psi ^2_{z}+u_{1y}-\frac{1}{3}\psi _{t}-\frac{1}{3}\psi _{z}\right) \psi _{yz}+\left( \frac{1}{3}\psi ^2_{y}+\left( \frac{5}{3}\psi _{t}-\frac{2}{3}\right) \psi _{y}+u_{1y}+\frac{5}{3}\psi ^2_{t}\right. \\ &{}\displaystyle \left. -\frac{1}{3}\psi ^2_{z}-\frac{5}{3}\psi _{t}-\frac{1}{3}\psi _{z}+\frac{1}{3}\right) \psi _{y}\psi _{tt}-\left( \frac{1}{3}\psi ^2_{t}+\frac{1}{3}\psi _{t}\psi _{y}-\frac{5}{3}\psi ^2_{z}+u_{1y}-\frac{1}{3}\psi _{t}-\frac{5}{3}\psi _{z}-\frac{1}{3}\right) \psi _{y}\psi _{zz}\\ &{}\displaystyle +\left( \frac{1}{2}\psi _{t}+\frac{1}{2}\psi _{z}-\frac{1}{2}\psi ^2_{t}+\frac{1}{2}\psi ^2_{z}-\frac{3}{2}u_{1y}+\frac{1}{2}\psi _{t}\psi _{y}\right) u_{1yy}-\frac{8}{3}\left( \left( \psi _{z}+\frac{1}{2}\right) \left( \psi _{t}+\frac{1}{2}\psi _{y}-\frac{1}{2}\right) \psi _{zt}\right. \\ &{}\displaystyle \left. \left. \left. +\left( -\frac{3}{4}\psi _{t}+\frac{3}{8}\psi _{y}+\frac{3}{8}\right) u_{1yt}+\left( \frac{3}{4}\psi _{z}+\frac{3}{8}\right) u_{1zy}+\frac{3}{4}\psi _{y}\left( u_{1tt}-u_{1zz}\right) \right) \psi _{y}\right) \psi _{y}\right) \end{array} \end{aligned}$$

where $u_1,u_4$ is free and $\psi =\psi (x,y,z,t)$. The compatibility condition does not hold at resonance $j=6$ is

$$\begin{aligned} \begin{array}{lll} \displaystyle &{}\displaystyle -6\left( \left( -\frac{4}{3}\left( \psi _{z}+\frac{1}{2}\right) \left( \psi _{t}+\frac{3}{4}\psi _{y}-\frac{1}{2}\right) \psi _{zt}+\left( \left( \psi _{t}-\frac{1}{2}\right) \psi _{y}+\frac{1}{6}+\psi ^2_{t}-\frac{1}{3}\psi ^2_{z}-\frac{1}{3}\psi _{z}+u_{1y}-\psi _{t}\right) \psi _{tt}\right. \right. \\ &{}\displaystyle \left. \left. +\left( \frac{1}{6}-\frac{1}{3}\psi ^2_{t}+\psi ^2_{z}+\frac{1}{3}\psi _{t}-u_{1yy}+\psi _{z}\right) \psi _{zz}+\left( \psi _{t}-\frac{1}{2}\right) u_{1yt}+\left( -\psi _{z}-\frac{1}{2}\right) u_{1yz}-\psi _{y}\left( u_{1tt}-u_{1zz}\right) \right) \psi _{yy}\right. \\ &{}\displaystyle +\left( \left( \frac{1}{2}-\psi _{t}\right) \psi _{y}+\frac{1}{3}\psi _{z}-u_{1y}-\psi ^2_{t}+\frac{1}{3}\psi ^2_{z}+\psi _{t}-\frac{1}{6}\right) \psi ^2_{yt}+\left( \frac{4}{3}\left( \psi _{z}+\frac{1}{2}\right) \left( \psi _{t}+\frac{3}{4}\psi _{y}-\frac{1}{2}\right) \psi _{yz}\right. \\ &{}\displaystyle \left. -\frac{2}{3}\left( \psi _{z}+\frac{1}{2}\right) \psi _{y}\psi _{zt}+\frac{2}{3}\left( \psi _{t}-\frac{3}{2}\psi _{y}-\frac{1}{2}\right) \psi _{y}\psi _{zz}+\left( \frac{1}{2}-\psi _{t}\right) u_{1yy}+u_{1yt}\psi _{y}\right) \psi _{yt}+\left( -\frac{1}{6}+\frac{1}{3}\psi ^2_{t}-\psi ^2_{z}\right. \\ &{}\displaystyle \left. -\frac{1}{3}\psi _{t}+u_{1y}-\psi _{z}\right) \psi ^2_{zy}+\left( -\frac{2}{3}\left( \psi _{t}-\frac{3}{2}\psi _{y}-\frac{1}{2}\right) \psi _{y}\psi _{zt}+\frac{2}{3}\left( \psi _{z}+\frac{1}{2}\right) \psi _{y}\psi _{tt}+\left( \psi _{z}+\frac{1}{2}\right) u_{1yy}-u_{1yz}\psi _{y}\right) \psi _{yz}\\ &{}\displaystyle \left. -\frac{4}{3}\psi ^2_{y}\left( \psi _{tt}\psi _{zz}-\psi ^2_{zt}\right) \right) \psi ^2_{y}=0.\\ \end{array} \end{aligned}$$

Briefly speaking, Eq. (3) does not pass Painlevé test, which means it is not integrable in Painlevé sense.

## Periodic solution of (3+1)-dimensional KP-Boussinesq equation

In this part, we will derive periodic solution for Eq. (3) with the help of Hirota’s bilinear method. Under the transformation $u=(\ln \phi )_{x}$, Eq. (3) becomes

6 $$\begin{aligned} \bigg (D^3_xD_y+D_tD_x+D_tD_y+D^2_t-D^2_z+D_xD_z\bigg )\phi \cdot \phi =0, \end{aligned}$$

where *D* is bilinear operator and it is defined by

$$\begin{aligned} D_\rho ^m D_\varrho ^n f(\rho ,\varrho )g(\rho ,\varrho ) =(\partial _\rho -\partial _{\rho '})^m(\partial _\varrho -\partial _{\varrho '})^n[f(\rho ,\varrho )g(\rho ',\varrho ')]\mid _{\rho =\rho ',\varrho =\varrho '}. \end{aligned}$$

A straightforward computation indicates (Eq. 6) has the following form

7 $$\begin{aligned} \begin{array}{lll} &{} \displaystyle -2\phi ^2_t+2\phi \phi _{tt}+2\phi ^2_z-2\phi \phi _{zz} -2\phi _t\phi _y+2\phi \phi _{yt}-2\phi _t\phi _x+2\phi \phi _{xt}\\ &{} \displaystyle +6\phi _{xy}\phi _{xx}-6\phi _x\phi _{xxy}-2\phi _y\phi _{xxx} +2\phi \phi _{xxxy}-2\phi _z \phi _x+2\phi \phi _{xz}=0.\\ \end{array} \end{aligned}$$

Assuming $\phi$ can be written as

8 $$\begin{aligned} \phi =e^{\chi _1}\left( \delta _1\cos [\chi _2]+\delta _2\sin [\chi _2]\right) +e^{\chi _3}\left( \delta _3\cos [\chi _4]+\delta _4\sin [\chi _4]\right) +\Lambda _1e^{2\chi _1}+\Lambda _2e^{2\chi _3}, \end{aligned}$$

where $\chi _i=p_ix+k_iy+\omega _it+l_iz(i=1,2,3,4).$ Based on anstaz (Eq. 8), diverse exact solutions can be constructed. Here, we only consider four typical cases under the condition of $p_1=p_3=p_4=k_2=0$. Substituting Eq. (8) into Eq. (7) and zeroing all the coefficients of $e,\sin ,\cos$, we have

9 $$\begin{aligned} \textit{Case 1}:\quad&\displaystyle l_1=\frac{-k_1 p_2^3+k_3 p_2^3+k_1 \omega _2-k_3 \omega _2+l_3 p_2}{p_2},\quad l_2=\frac{\omega _2 \left( p_2+\omega _2\right) }{p_2},\quad l_4=\frac{k_4 \omega _2-k_4 p_2^3}{p_2},\nonumber \\&\displaystyle \omega _3=\omega _1,\quad \omega _4=0,\quad \Lambda _1=0,\quad \Lambda _2=0, \end{aligned}$$

10 $$\begin{aligned} \textit{Case 2}:\quad&\displaystyle l_1=\frac{-k_1 p_2^3+k_3 p_2^3-k_1 p_2+k_3 p_2-k_1 \omega _2+k_3 \omega _2+l_3 p_2}{p_2},\quad l_2=\frac{\omega _2 \left( p_2+\omega _2\right) }{p_2},\nonumber \\&\displaystyle l_4=-\frac{k_4 \left( p_2^3+p_2+\omega _2\right) }{p_2},\quad \omega _3=k_1-k_3+\omega _1,\quad \omega _4=-k_4,\quad \Lambda _1=0,\quad \Lambda _2=0, \end{aligned}$$

11 $$\begin{aligned} \textit{Case 3}:\quad&\displaystyle l_1=\frac{k_1 \omega _2-k_1 p_2^3}{p_2},\quad l_2=\frac{\omega _2 \left( p_2+\omega _2\right) }{p_2},\quad l_3=\frac{k_3 \omega _2-k_3 p_2^3}{p_2},\quad l_4=\frac{k_4 \omega _2-k_4 p_2^3}{p_2},\nonumber \\&\displaystyle \omega _1=0,\quad \omega _3=0,\quad \omega _4=0,\quad \Lambda _1=0, \end{aligned}$$

12 $$\begin{aligned} \textit{Case 4}:\quad&\displaystyle l_1=-\frac{p_2^3 \left( -\omega _1\right) -p_2 \omega _1-\omega _1 \omega _2}{p_2},\quad l_2=-\frac{-p_2 \omega _2-\omega _2^2}{p_2},\quad l_3=-\frac{p_2^3 \left( -\omega _3\right) -p_2 \omega _3-\omega _2 \omega _3}{p_2},\nonumber \\&\displaystyle l_4=-\frac{p_2^3 \left( -\omega _4\right) -p_2 \omega _4-\omega _2 \omega _4}{p_2},\quad k_1=-\omega _1,\quad k_3=-\omega _3,\quad k_4=-\omega _4,\quad \Lambda _2=0. \end{aligned}$$

**Figure 1:**
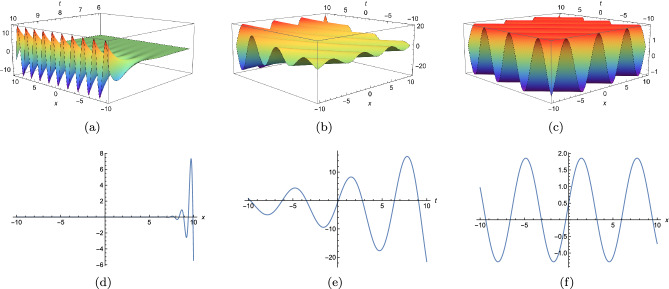
Three typical periodic solutions of Eq. (8) with $y=z=0$, (**a,d**) condition (2.3) under $\delta _1=\delta _4=2,\delta _2=\delta _3=1,\omega _1=2,\omega _2=3,\omega _3=1/2,k_1=k_4=1,k_3=2,p_2=3,$ (**b,e**) condition (2.3) under $\delta _1=1/2,\delta _2=\delta _3=1,\delta _4=2,\omega _2=1,k_1=1,k_3=k_4=2,p_2=1,$ (**c,f**) condition (10) under $\delta _1=1/2,\delta _2=\delta _3=1,\delta _4=2,\omega _2=1,k_1=1,k_3=k_4=2,p_2=1$.

Substituting (9)–(12) and $\chi _i=p_ix+k_iy+\omega _it+l_iz$ back to $u=(\ln \phi )_{x}$, we can derive periodic solutions for Eq. (3). Here, by choosing appropriate parameters, we have plotted three typical periodic solutions in Fig. 1, under conditions (9) and (10). As we can see, the first periodic solution is periodic along *x*-axis, while the third one is periodic in each direction(both *x*- and *t*-axis). As regards the middle one, based on Fig. 1e, the period decreases while the amplitude tends to become greater along *t*-axis.

## Multi-soliton and shock wave of (3+1)-dimensional KP-Boussinesq equation

Starting with Burgers equation

13 $$\begin{aligned} \mu _t-\mu _{xx}-2\mu \mu _x=0. \end{aligned}$$

Setting $\mu =\mathcal {T}(\mathcal {\gamma })$, $\gamma =px+ky+lz-\omega t$, Eq. (13) turns to

14 $$\begin{aligned} -\omega \mathcal {T}-p^2 \mathcal {T}_{\gamma }-p \mathcal {T}^2=0, \end{aligned}$$

with the multi-soliton solution^36^

15 $$\begin{aligned} \mathcal {T}=\frac{\sum ^m_{j=1} p_j e^{p_j x-\omega _j t+x_{j,0}}}{1+\sum ^m_{j=1} e^{p_j x-\omega _j t+x_{j,0}}}, \end{aligned}$$

where $x_{j,0}$ is constant, $\omega _j=-k^2_j.$ Back to Eq. (3) with the transformation $u(x,y,z,t)=\mathcal {R}(\gamma )$, we have

16 $$\begin{aligned} \left( -p l+\omega ^2-\omega p-\omega k\right) \mathcal {R}_{\gamma }+3p^2k \mathcal {R}^2_{\gamma }+p^3k\mathcal {R}_{\gamma \gamma \gamma }=0. \end{aligned}$$

Balancing the highest order nonlinear term and partial term, we have

17 $$\begin{aligned} \mathcal {R}(\gamma )=\mathcal {J}_0+\mathcal {J}_1\mathcal {T}(\gamma ), \end{aligned}$$

where $\mathcal {J}_0,\mathcal {J}_1$ are constants. Substituting Eqs. (17) and (14) into Eq. (16) results an algebraic system

$$\begin{aligned} \begin{array}{lll} &{} \displaystyle 3 J_1^2 k-6 J_1 k=0,\\ &{} \displaystyle 12 J_1 k p-6 J_1^2 k p=0,\\ &{} \displaystyle 3 J_1^2 k p^2-7 J_1 k p^2+\frac{J_1 k \omega }{p}+J_1 l-\frac{J_1 \omega ^2}{p}+J_1 \omega =0,\\ &{} \displaystyle J_1 k p^3-J_1 k \omega -J_1 l p-J_1 p \omega +J_1 \omega ^2=0.\\ \end{array} \end{aligned}$$

Solving above system, we have

$$\begin{aligned} p^3-\omega \ne 0,\quad p\ne 0,\quad k=\frac{l p+p \omega -\omega ^2}{p^3-\omega },\quad J_1=2. \end{aligned}$$

Therefore, the *n*-soliton solution of Eq. (3) reads

18 $$\begin{aligned} \displaystyle u=\mathcal {J}_0+2\frac{\displaystyle \sum _{j=1}^m \frac{\left( -l_j p_j+p_j \omega _j-\omega _j^2\right) \exp \left( p_j x+\frac{\left( -l_j p_j+p_j \omega _j-\omega _j^2\right) }{p_j^3-\omega _j}y+ l_j z -\omega _j t+x_{j,0}\right) }{p_j^3-\omega _j}}{\displaystyle 1+\sum _{j=1}^m \exp \left( p_j x+\frac{\left( -l_j p_j+p_j \omega _j-\omega _j^2\right) }{p_j^3-\omega _j}y+l_j z-\omega _j t+x_{j,0}\right) } \end{aligned}$$

with the constraint $p_j^3-\omega _j \ne 0,p_j\ne 0.$

To construct multi-soliton solutions to nonlinear evolution equations, Hirota developed a new “direct method” in 1971^5^. The goal was to transform existing variables into new ones so that multi-soliton solutions would display particularly straightforwardly. The equations were found to be quadratic for the new dependent variables, and all derivatives showed up as Hirota’s bilinear derivatives (hence the name “Hirota bilinear form”). Although inspired by the inverse scattering transform (IST), the Hirota’s direct method does not need strong, sophisticated assumptions like the IST and applies to a wider range of equations than the IST. All of which makes Hirota’s direct method the optimal tool to understand soliton scattering for further development of soliton theory. The multi-soliton solutions obtained by the Hirota’s direct method through the Hirota bilinear form, also known as *n*-soliton solution, has the form

$$\begin{aligned} f=\displaystyle \sum \exp \left[ \sum \limits ^{N}_{i=1} \mu _i \eta _i+\sum \limits ^{(N)}_{i<j}A_{ij} \mu _i \mu _j\right] , \end{aligned}$$

where the first $\sum$ means a summation over all possible combinations of $\mu _i=0,1$($i=1,...,N$). $\sum \limits ^{(N)}_{i<j}$ means a summation over all possible pairs (*i*, *j*) chosen from $\{1,2,...,N\}$ for $i<j.$

**Figure 2:**
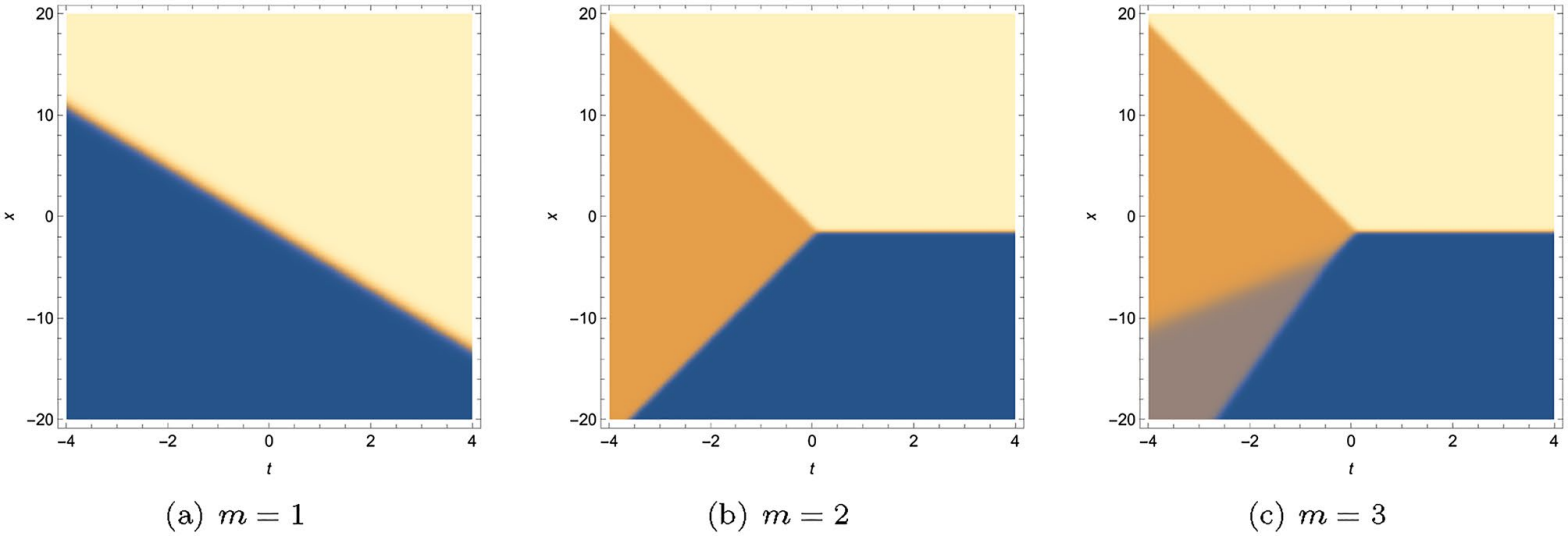
Density profile of Eq. (15) with $y=z=0$, (**a**) $p_1=1,\omega _1=-3,x_{1,0}=1$, (**b**) $p_1=p_2=1,\omega _1=-\omega _2=-5,x_{1,0}=1,x_{2,0}=2$, (**c**) $p_1=p_2=p_3=1,\omega _1=-\omega _2=-5,\omega _3=2,x_{1,0}=1,x_{2,0}=2,x_{3,0}=3.$.

**Figure 3:**
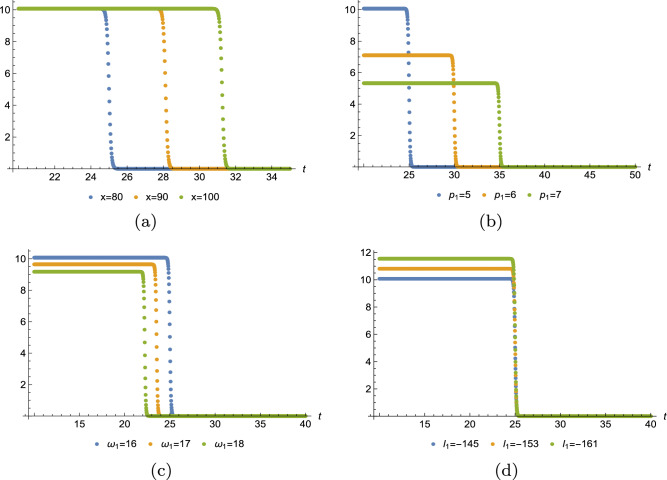
Sectional profile of Eq. (18) with $m=1$,$\mathcal {J}_0=y=z=0$, $x_{j,0}=0$, (**a**) $p_1=5,\omega _1=16,l_1=-725/5$, (**b**) $\omega _1=16,l_1=-725/5,x=80$, (**c**) $p_1=5,l_1=-725/5,x=80$, (**d**) $p_1=5,\omega _1=16,x=80$.

**Figure 4:**
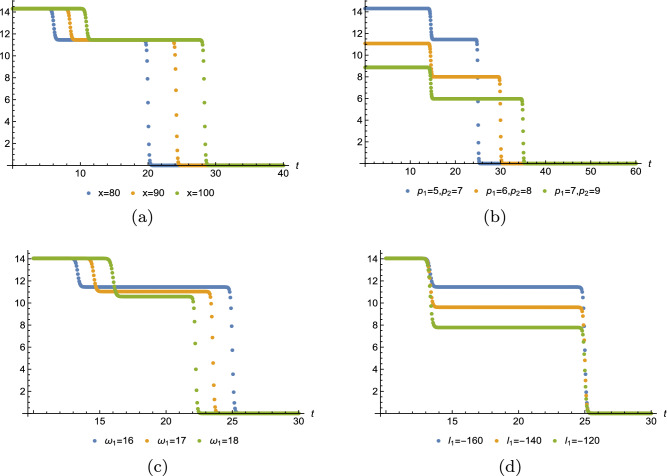
Sectional profile of Eq. (18) with $m=2$,$\mathcal {J}_0=y=z=0$, $x_{j,0}=0$, (**a**) $p_1=5,p_2=7,\omega _1=16,\omega _2=27,l_1=-800/5,l_2=-2800/7$, (**b**) $\omega _1=16,\omega _2=27,l_1=-800/5,l_2=-2800/7,x=80$, (**c**) $p_1=5,p_2=7,\omega _2=28,l_1=-800/5,l_2=-2800/7,x=80$, (**d**) $p_1=5,p_2=7,\omega _1=16,\omega _2=28,l_2=-2800/7,x=80$.

**Figure 5:**
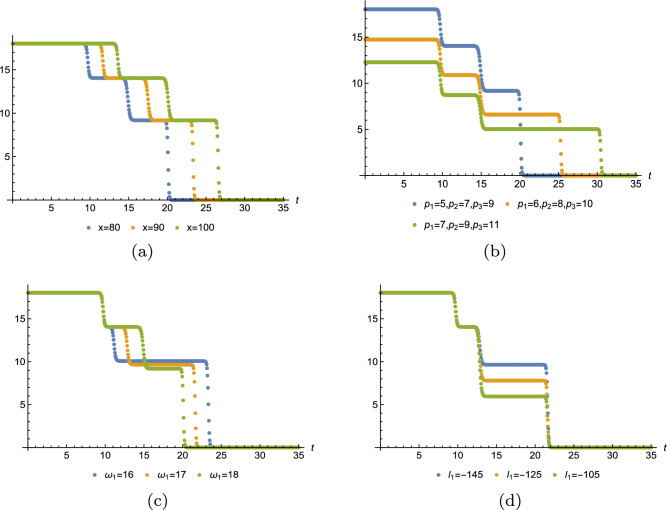
Sectional profile of Eq. (18) with $m=3$,$\mathcal {J}_0=y=z=0$, $x_{j,0}=0$, (**a**) $p_1=5,p_2=7,p_3=9,\omega _1=18,\omega _2=27,\omega _3=39,l_1=-725/5,l_2=-2760/7,l_3=-821$, (**b**) $\omega _1=18,\omega _2=27,\omega _3=39,l_1=-725/5,l_2=-2760/7,l_3=-821,x=80$, (**c**) $p_1=5,p_2=7,p_3=9,\omega _2=27,\omega _3=39,l_1=-725/5,l_2=-2760/7,l_3=-821,x=80$, (**d**) $p_1=5,p_2=7,p_3=9,\omega _1=17,\omega _2=27,\omega _3=39,l_2=-2760/7,l_3=-821,x=80$.

Below, we have plotted the one-, two-, three-soliton solution of Eq. (15). Figure 2 displays one, three, four divided waves, separately. Moreover, it is possible to construct any number of waves for Eq. (3) with Eq. (18) and diverse patterns of the wave can be obtained in terms of manipulating the value of $x,p_i,\omega _1,l_1.$ For instance, Fig. 3 demonstrates one-shock wave with different values of $x,p_1,\omega _1,l_1.$ Numerical evidence indicates that the increase of *x* makes the wave travel longer in *t*-axis and $l_1$ influences the amplitude of the wave, the greater $|l_1|$, the higher amplitude. For $p_1$ and $\omega _1$, they affect both the distance and the amplitude, but quite different effect. As shown in Fig. 3b, as $p_1$ grows, the wave moves along the *t*-axis and the amplitude decreases. In Fig. 3c, as $\omega _1$ grows, the wave moves towards the negative direction of *t*-axis and the amplitude decreases. Figs. 4 and 5 illustrate two- and three-shock waves with different values of $x,p_1,p_2,\omega _1,l_1$ and $x,p_1,p_2,p_3,\omega _1,l_1.$ These parameters function just the same as in Fig. 3.

## Variable separation solution of (3+1)-dimensional KP-Boussinesq equation

As is address in the introduction, we have proposed a new variable separation solution

$$\begin{aligned} u=\lambda _1 \digamma _1[x]+\lambda _2 \digamma _2[y]+\lambda _3 \digamma _3[z]+\lambda _4 \digamma _4[t], \end{aligned}$$

where $\lambda _i(i=1,...,4)$ are constants to be determined later, $\digamma _1[x],\digamma _2[y],\digamma _3[z],\digamma _4[t]$ are free functions. Next, we will use it to derive the folded localized excitation for Eq. (3). Substitution into Eq. (3), one has

$$\begin{aligned} \digamma _1[x]=k_1x+b_1,\quad \digamma _4[t]=k_4t+b_4. \end{aligned}$$

where $k_1,b_1,k_4,b_4$ are constants and $\digamma _2,\digamma _3$ are free functions. Thus, Eq. (3) has following solution

19 $$\begin{aligned} u=\lambda _1 (k_1x+b_1)+\lambda _2 \digamma _2[y]+\lambda _3 \digamma _3[z]+\lambda _4 (k_4t+b_4). \end{aligned}$$

### Folded solitary wave

**Figure 6:**
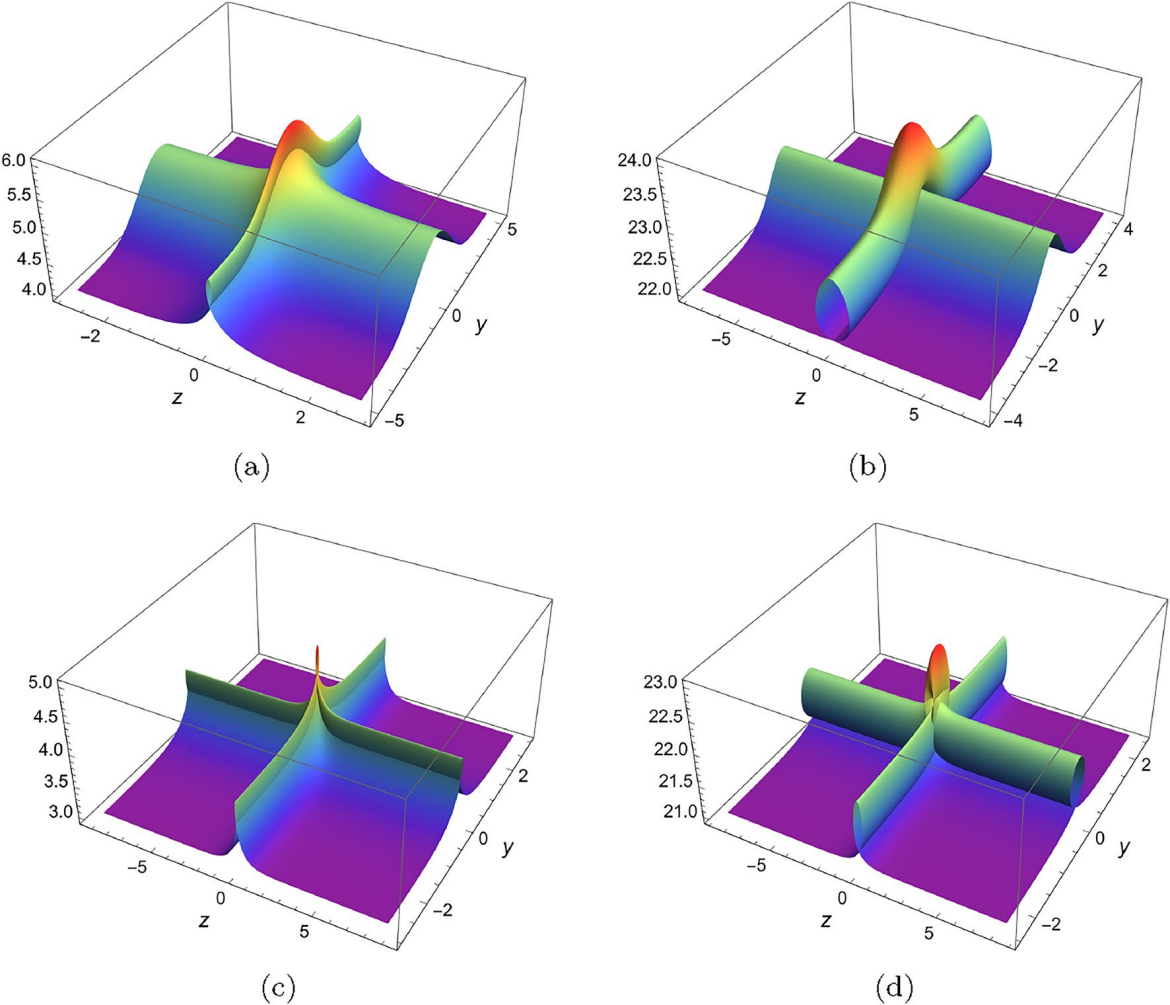
Variable separation solution (19) of Eq. (3) with $\lambda _1=\lambda _2=\lambda _3=\lambda _4=1,k_1=b_1=k_4=b_4=1$, $\digamma _2=\textrm{sech}^2(\xi ),\digamma _3=\textrm{sech}^2(\theta )$, $x=t=0$, (**a**) $z=\xi -\textrm{tanh}[\xi ],y=\theta +\textrm{tanh}[\theta ]$, (**b**) $z=\xi -2.5\textrm{tanh}[\xi ],y=\theta$, (**c**) $z=\xi -1.15\textrm{tanh}[\xi ],y=\theta -1.15\textrm{tanh}[\theta ]$, (**d**) $z=\xi -1.6\textrm{tanh}[\xi ],y=\theta -1.6\textrm{tanh}[\theta ]$.

To establish folded localized excitations of *u*, it is essential using the freedom of $\digamma _2,\digamma _3$, and set them to be suitable multi-valued functions. For example, set

20 $$\begin{aligned} \begin{array}{lll} &{}\displaystyle \digamma _2=\sum ^{M}_{i=1}\varphi _i[\xi ],\\ &{}\displaystyle y=\xi +\sum ^{M}_{i=1}\psi _i[\xi ],\\ \end{array} \end{aligned}$$

where $\varphi _i,\psi _i$ are localized excitations($\varphi _i(\pm \infty )=\psi _i(\pm \infty )=constant$). One can make $\xi$ multi-valued by taking $\psi _i$ wisely in some regions of *y*. Hence, $\digamma _2$ is *M* localized excitations as $\xi |_{y\rightarrow \infty }\rightarrow \infty$ and may be multi-valued of *y* even if it is single-valued of $\xi$. Actually, most of the multi-loop solutions are the special cases of Eq. (20). Based on above manner, first we choose $\digamma _2=\textrm{sech}^2[\xi ],\digamma _3=\textrm{sech}^2[\theta ]$ and set *z*, *y* to be

$$\begin{aligned} \begin{array}{lll} &{}\displaystyle z=\xi +\alpha \textrm{tanh}[\xi ],\\ &{}\displaystyle y=\theta +\beta \textrm{tanh}[\theta ],\\ \end{array} \end{aligned}$$

where $\alpha$ and $\beta$ are used to regulate the shape of the foldon. Below, we have plotted four types folded solitary wave in (*y*, *z*)-plane, by setting $\alpha =-1.2,\beta =1$; $\alpha =-2.5,\beta =0$; $\alpha =-1.5,\beta =-1.5$; $\alpha =-1.6,\beta =-1.6$. As we can see in Fig. 6a, b, there are two waves, one wave overlies another. While in Fig. 6c, d, two pipe-shaped solitary waves interact at (0, 0) and a peak rises at the intersection point.

**Figure 7:**
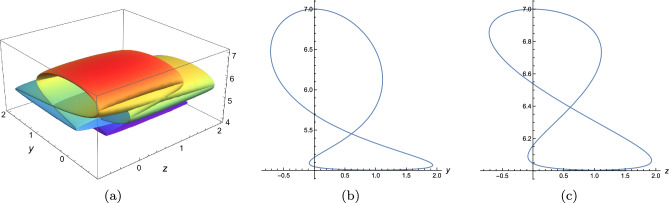
Variable separation solution (19) of Eq. (3) with $\lambda _1=\lambda _2=\lambda _3=\lambda _4=1,k_1=b_1=k_4=b_4=1$, $\digamma _2=\textrm{sech}^2(\xi ),\digamma _3=\textrm{sech}^2(\theta )+\textrm{sech}^6(\theta )$, $x=t=0$, $z=\xi +2\textrm{tanh}[\xi ]+\textrm{tanh}^2[\xi ]-5.5\textrm{tanh}^3[\xi ],y=\theta +2\textrm{tanh}[\theta ]+\textrm{tanh}^2[\theta ]-5.5\textrm{tanh}^3[\theta ]$, (**a**) 3D plot, (**b**) sectional view along z-axis, (**c**) sectional view along y-axis.

**Figure 8:**
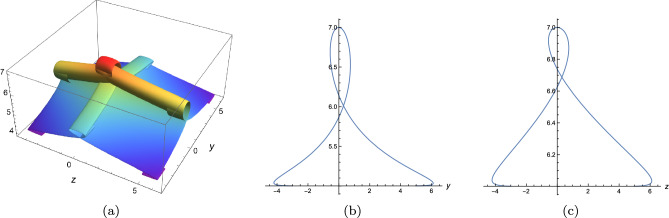
Variable separation solution (19) of Eq. (3) with $\lambda _1=\lambda _2=\lambda _3=\lambda _4=1,k_1=b_1=k_4=b_4=1$, $\digamma _2=\textrm{sech}^2(\xi ),\digamma _3=\textrm{sech}^2(\theta )+\textrm{sech}^6(\theta )$, $x=t=0$, $z=\xi +2\textrm{tanh}[\xi ]+\textrm{tanh}^2[\xi ]-10\textrm{tanh}^3[\xi ],y=\theta +2\textrm{tanh}[\theta ]+\textrm{tanh}^2[\theta ]-10\textrm{tanh}^3[\theta ]$, (**a**) 3D plot, (**b**) sectional view along z-axis, (**c**) sectional view along y-axis.

Secondly, we choose $\digamma _2=\textrm{sech}^2[\xi ],\digamma _3=\textrm{sech}^2[\theta ]+\textrm{sech}^6[\theta ]$ and set *z*, *y* to be

$$\begin{aligned} \begin{array}{lll} &{}\displaystyle z=\xi +\alpha \textrm{tanh}[\xi ]+\beta \textrm{tanh}^2[\xi ]+\gamma \textrm{tanh}^3[\xi ],\\ &{}\displaystyle y=\theta +\alpha \textrm{tanh}[\theta ]+\beta \textrm{tanh}^2[\theta ]+\gamma \textrm{tanh}^3[\theta ],\\ \end{array} \end{aligned}$$

where $\alpha ,\beta ,\gamma$ are free parameters. In Fig. 7, the parameters are chosen to be $\alpha =2,\beta =1,\gamma =-5.5$ and it shows a picture of superimposed multi-foldon. The sectional views Fig. 7b, c are plotted along *y*- and *z*-axis and demonstrate two-loop structure. Fig. 8 is plotted by choosing $\alpha =2,\beta =1,\gamma =-10$.

**Figure 9:**
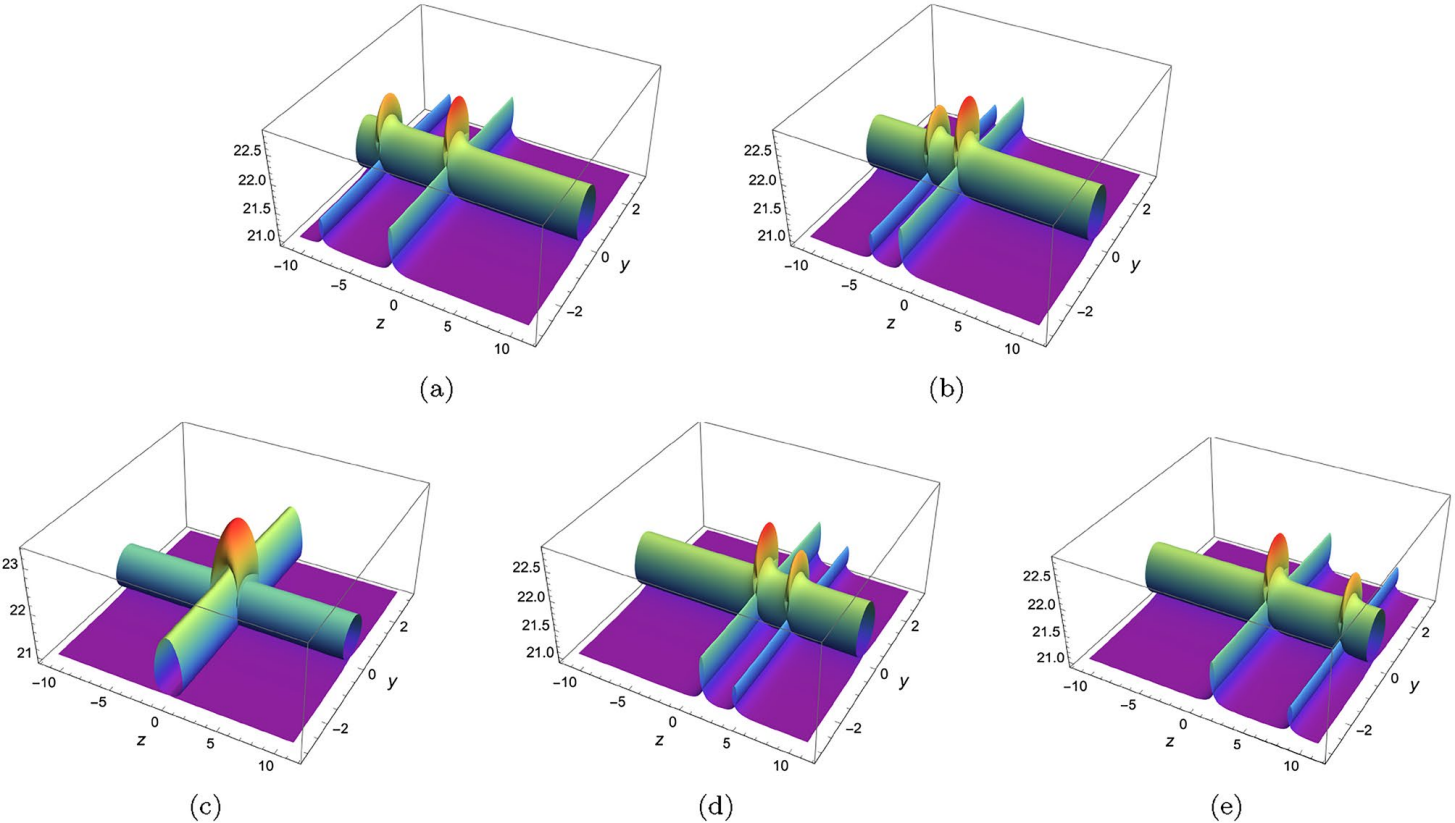
Evolution progress of Eq. (19) with $\lambda _1=\lambda _2=\lambda _3=\lambda _4=1,k_1=b_1=k_4=b_4=1$, $\digamma _2=\frac{4}{5}\textrm{sech}^2[\xi ]+\frac{1}{2}\textrm{sech}^2[\xi +C], \digamma _3=\textrm{sech}^2[\theta ]$, $x=t=0$, $z=\xi -1.5\textrm{tanh}[\xi ]-1.5\textrm{tanh}[\xi -C],y=\theta -2\textrm{tanh}[\theta ]$, (**a**) $C=-10$, (**b**) $C=-6$, (**c**) $C=0$, (**d**) $C=6$, (**e**) $C=10$.

**Figure 10:**
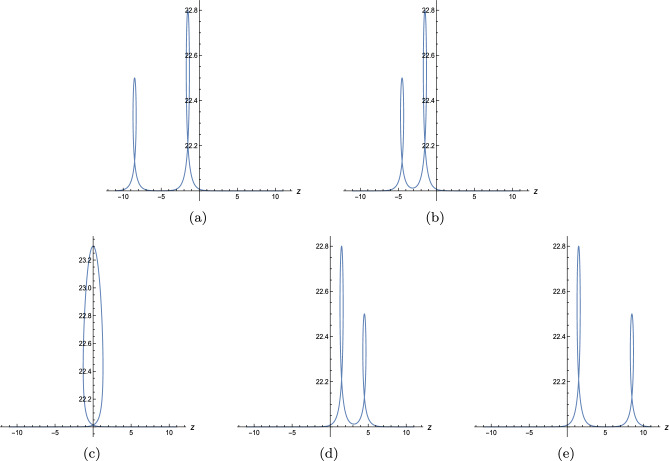
Corresponding sectional view of Fig. 9 at $y=0$.

In addition, we introduce a free parameter to simulate the evolution behaviour of the foldons. Here, $\digamma _2,\digamma _3$ are set to be

$$\begin{aligned} \begin{array}{lll} &{}\displaystyle \digamma _2=\frac{4}{5}\textrm{sech}^2[\xi ]+\frac{1}{2}\textrm{sech}^2[\xi +C],\quad \digamma _3=\textrm{sech}^2[\theta ],\\ &{}\displaystyle z=\xi -1.5\textrm{tanh}[\xi ]-1.5\textrm{tanh}[\xi -t],\quad y=\theta -2\textrm{tanh}[\theta ].\\ \end{array} \end{aligned}$$

where *C* is free parameter used to regulate the position, ranging from $-10$ to 10. For convenience, we call it “time”. As we can see in Figs. 9 and  10, it depicts the chase-after phenomenon between one pipe-shaped foldon and two stripe foldon. Two stripe foldons move along the positive *z*-direction while the pipe-shaped foldon keeps still, crossing the stripe foldons. The shorter stripe foldon travels faster than the bigger one and they meet with each other at the moment $C=0$(see Figs. 9c,  10c). Here, they converge into a single large pipe-shaped foldon that has the maximum amplitude. The fact that Fig. 10a, e, as well as Fig 10b, d, are exactly the same image in reverse order indicates that the collision is elastic.

### Superimposed structure of folded solitary wave

**Figure 11:**
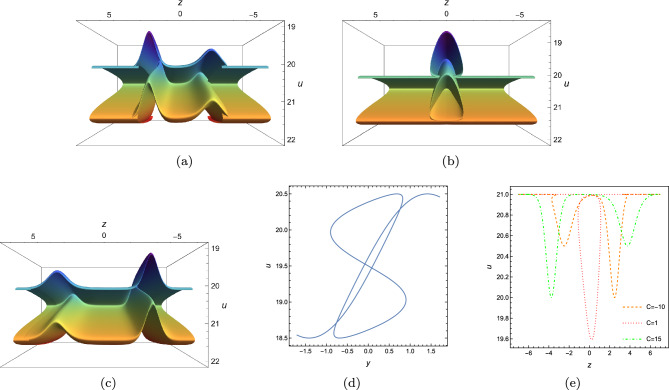
Evolution progress of two foldons of Eq. (19) with $\lambda _1=\lambda _2=\lambda _3=\lambda _4=1,k_1=b_1=k_4=b_4=1$, $\digamma _2=-\textrm{sech}^4[\xi +0.25C]-0.5\textrm{sech}^2[\xi -0.25C], \digamma _3=-\sin [\theta ]$, $x=t=0$, $z=\xi -\mu \textrm{tanh}[\xi -0.5C]-\gamma \textrm{tanh}[\xi +0.5C],y=\theta +2\textrm{tanh}[\theta ]-5.3\textrm{tanh}^3[\theta ]$, (**a**) $C=-10$, (**b**) $C=0$, (**c**) $C=15$, (**d**) sectional view at $z=0,C=0$, (**e**) sectional view at $y=0,C=-10,1,15$.

In this section we will consider superimposed structure of folded solitary wave and analyze its corresponding dynamical behaviour. Firstly, let us restrain $\digamma _2,\digamma _3$ as

$$\begin{aligned} \begin{array}{lll} &{}\displaystyle \digamma _2=-\textrm{sech}^4[\xi +0.25C]-0.5\textrm{sech}^2[\xi -0.25C],\quad \digamma _3=-\sin [\theta ],\\ &{}\displaystyle z=\xi -\mu \textrm{tanh}[\xi -0.5C]-\gamma \textrm{tanh}[\xi +0.5C],\quad y=\theta +2\textrm{tanh}[\theta ]-5.3\textrm{tanh}^3[\theta ],\\ \end{array} \end{aligned}$$

where $\gamma ,\mu$ are free parameters, $\theta ,\xi$ are intermediate variables, *C* is a constant used to control evolution. Based on above expression, the evolutional plots are shown in Fig. 11 under the parametric selection: $C=-10,0,15$ and $\mu =\gamma =1.5.$ It models the interaction between two folded solitary waves and each of them is double-bell-shaped. Although the sectional view along *y*-axis is single-bell-shaped(see Fig. 11e), it is worth noting that the sectional view along *z*-axis displays a more complicated multi-valued structure(see Fig. 11d) and it may maps up to five values for some values of *y*(see the interval near $y=-0.5$). As we can see, there are two double-bell-shaped foldons(one shorter and one higher) at $C=-10$. As *C* increases, two foldons integrate into a much bigger one foldon. After that, it divides into two. It is noteworthy that readers can found that the sectional plots do not match 3D plots since we actually draw the anti-folded solitary waves and reverse the pictures.

Moreover, we select four different combinations of $(\mu ,\gamma )$: $\mu =0.9,\gamma =0.1$, $\mu =\gamma =0.1$, $\mu =\gamma =0.9$, $\mu =\gamma =1.5$ to investigate the superimposed structures of two folded solitary waves and *y* is chosen to be

$$\begin{aligned} y=\theta +2\textrm{tanh}^2[\theta ]-5.3\textrm{tanh}^3[\theta ]. \end{aligned}$$

**Figure 12:**
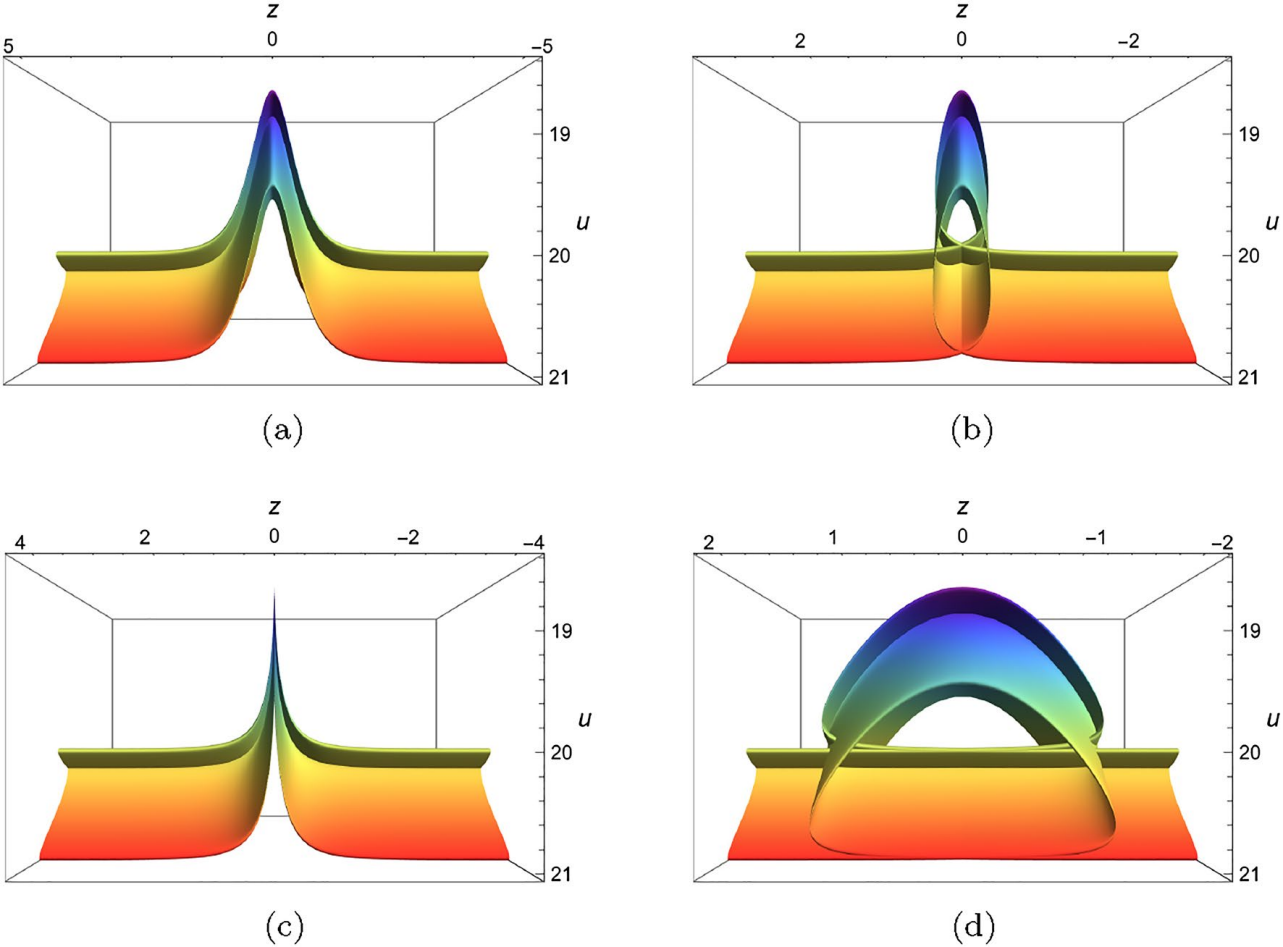
Interaction of two foldons of Eq. (19) with $\lambda _1=\lambda _2=\lambda _3=\lambda _4=1,k_1=b_1=k_4=b_4=1$, $\digamma _2=-\textrm{sech}^4[\xi +0.25C]-0.5\textrm{sech}^2[\xi -0.25C], \digamma _3=-\textrm{sech}^2[\theta ]$, $C=x=t=0$, $z=\xi -\mu \textrm{tanh}[\xi -0.5C]-\gamma \textrm{tanh}[\xi +0.5C],y=\theta +2\textrm{tanh}^2[\theta ]-5.3\textrm{tanh}^3[\theta ]$ (**a**) $\mu =\gamma =0.1$, (**b**) $\mu =\gamma =0.9$, (**c**) $\mu =0.9,\gamma =0.1$, (**d**) $\mu =\gamma =1.5$.

As shown in Fig. 12, four pictures demonstrate their interaction states at $C=0$, and can be classified as tent-shaped structure (Fig.  12a), peak-shaped structure(Fig. 12c), loop-shaped structure(Fig. 12b, d).

**Figure 13:**
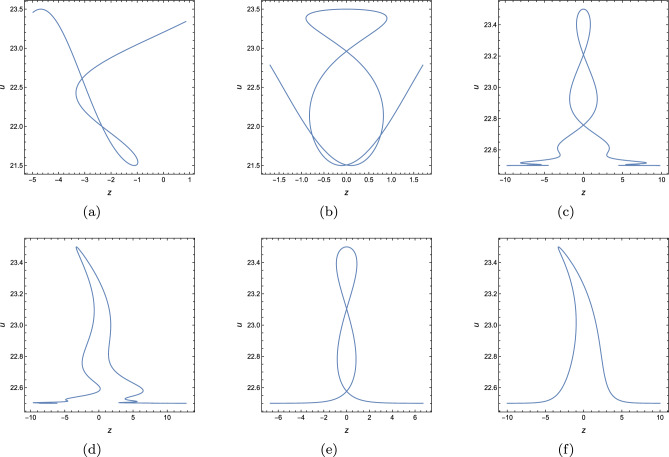
Sectional profile with with $\lambda _1=\lambda _2=\lambda _3=\lambda _4=1,k_1=b_1=k_4=b_4=1$, $\digamma _2=\textrm{sech}^4[\xi +0.25C]+0.5\textrm{sech}^2[\xi -0.25C]$, $x=t=0$, $z=C=0$, (**a**) $\digamma _3=1.5\sin [\theta ],y=\theta +3\textrm{sech}[\theta ]-7\textrm{sech}^3[\theta ]$, (**b**) $\digamma _3=1.5\cos [\theta ],y=\theta +3\textrm{tanh}[\theta ]-7\textrm{tanh}^3[\theta ]$, (**c**) $\digamma _3=1.5\textrm{sech}[\theta ],y=\theta +3\sin [\theta ]-7\sin ^3[\theta ]$, (**d**) $\digamma _3=1.5\textrm{sech}[\theta ],y=\theta +3\cos [\theta ]-7\cos ^3[\theta ]$, (**e**) $\digamma _3=1.5\textrm{sech}[\theta ],y=\theta +3\textrm{tanh}[\theta ]-7\textrm{tanh}^3[\theta ]$, (**f**) $\digamma _3=1.5\textrm{sech}[\theta ],y=\theta +3\textrm{sech}[\theta ]-7\textrm{sech}^3[\theta ]$.

Multi-valued structure Fig. 11d provides us an inspiration to derive other pattern. Motivated by such idea, we pay attention to 2D geometric plot in (*z*, *u*)-plane under the selections: $\digamma _2=-\textrm{sech}^4[\xi +0.25C]-0.5\textrm{sech}^2[\xi -0.25C]$ and $\digamma _3=1.5\sin [\theta ],y=\theta +3\textrm{sech}[\theta ]-7\textrm{sech}^3[\theta ]$; $\digamma _3=1.5\cos [\theta ],y=\theta +3\textrm{tanh}[\theta ]-7\textrm{tanh}^3[\theta ]$; $\digamma _3=1.5\textrm{sech}[\theta ],y=\theta +3\sin [\theta ]-7\sin ^3[\theta ]$; $\digamma _3=1.5\textrm{sech}[\theta ],y=\theta +3\cos [\theta ]-7\cos ^3[\theta ]$; $\digamma _3=1.5\textrm{sech}[\theta ],y=\theta +3\textrm{tanh}[\theta ]-7\textrm{tanh}^3[\theta ]$; $\digamma _3=1.5\textrm{sech}[\theta ],y=\theta +3\textrm{sech}[\theta ]-7\textrm{sech}^3[\theta ]$. As shown in Fig. 13, six novel structures have been obtained and at least of one variable of *z* will map into more than one value. These graphics can be sorted out as: double-loop pattern(Fig. 13c, e), anti-twisted double-loop pattern(Fig. 13a), anti-umbrella pattern(Fig. 13b), fin-shape pattern(Fig. 13d, f).

## Summary

In this work, a new (3+1)-dimensional KP-Boussinesq equation is put forward and investigated, which describes the long wavelength water wave in hydrodynamics. Firstly, we have derived periodic solutions in terms of Hirota’s bilinear form and ansatz, under certain condition. Three typical periodic solutions have been plotted in 2D and 3D, see Fig. 1. Secondly, taking Burgers equation as an auxiliary function, we have obtained *n*-soliton solution as well as *n*-shock wave. In addition, their mechanism has been systematically discussed by exploring the function of each parameter, see Figs. 2, 3, 4 and 5. Last but not least, we have offered a brand new insight into deriving multi-valued soliton from (3+1)-dimensional and higher dimensional partial differential equation, and use it to establish folded localized excitation of Eq. (3). Moreover, we have studied the superimposed structures and interaction behaviours of folded solitary waves. A lot of novel structures have been constructed, see Figs. 6, 7, 8, 9, 10, 11, 12 and  13. Compared with the other methods, the variable separation solutions obtained in this paper not only directly give the analytical form of the solution *u* instead of its potential $u_y$, but also provide us a straightforward approach to construct localized excitation for higher order dimensional nonlinear partial differential equation. We believe the method given in current paper will be helpful in finding soliton and localized excitation solution for other nonlinear systems.

## Data Availability

The datasets used and/or analysed during the current study available from the corresponding author on reasonable request.
